# Adolescents’ Psychological Consequences and Cyber Victimization: The Moderation of School-Belongingness and Ethnicity

**DOI:** 10.3390/ijerph16142493

**Published:** 2019-07-12

**Authors:** Michelle F. Wright, Sebastian Wachs

**Affiliations:** 1 Department of Psychology, Pennsylvania State University, State College, PA 16802, USA; 2 Faculty of Social Studies, Masaryk University, 60200 Brno, Czech Republic; 3 Department of Educational Studies, University of Potsdam, 14476 Potsdam, Germany

**Keywords:** cyberbullying, cyber victimization, depression, anxiety, loneliness, Latinx, Latino, adolescents, ethnic, ethnic differences

## Abstract

Cyber victimization research reveals various personal and contextual correlations and negative consequences associated with this experience. Despite increasing attention on cyber victimization, few studies have examined such experiences among ethnic minority adolescents. The purpose of the present study was to examine the moderating effect of ethnicity in the longitudinal associations among cyber victimization, school-belongingness, and psychological consequences (i.e., depression, loneliness, anxiety). These associations were investigated among 416 Latinx and white adolescents (46% female; *M* age = 13.89, *SD* = 0.41) from one middle school in the United States. They answered questionnaires on cyber victimization, school belongingness, depression, loneliness, and anxiety in the 7th grade (Time 1). One year later, in the 8th grade (Time 2), they completed questionnaires on depression, loneliness, and anxiety. Low levels of school-belongingness strengthened the positive relationships between cyber victimization and Time 2 depression and anxiety, especially among Latinx adolescents. The positive association between cyber victimization and Time 2 loneliness was strengthened for low levels of school-belongingness for all adolescents. These findings may indicate that cyber victimization threatens adolescents’ school-belongingness, which has implications for their emotional adjustment. Such findings underscore the importance of considering diverse populations when examining cyber victimization.

## 1. Introduction

Linked to depression, anxiety, loneliness, and school problems, cyber victimization has gained increased attention among educators, parents, and researchers [1,2]. It is currently unknown whether experiencing cyber victimization influences adolescents’ school-belongingness. Furthermore, with the increasing diversity in the U.S. population, along with the decline in the gaps regarding technology and internet among white and ethnic minority adolescents, another important direction in this research is to investigate ethnicity differences in the psychological consequences of cyber victimization. Including ethnicity as a moderator in the associations among psychological consequences (i.e., depression, anxiety, loneliness), cyber victimization, and school-belongingness is important as minority adolescents generally report lower school-belongingness [3]. Therefore, perceiving less school-belongingness may strengthen the positive relationship between cyber victimization and psychological consequences, especially among ethnic minority adolescents. To this end, the present study examined ethnicity differences (i.e., white, Latinxs) in the associations among cyber victimization, school-belongingness, and psychological consequences of cyber victimization.

### 1.1. Cyber Victimization and Adjustment Difficulties

Cyber victimization is defined as being intentionally harmed through various behaviors (e.g., spreading rumors, hacking someone’s accounts, sending degrading messages) deemed to be offensive, derogatory, and unwanted [4,5,6]. These behaviors are experienced online and through information and communication technologies (ICTS), such as social networking sites, email, chat programs, text messages, and gaming consoles. The behaviors used to harm others online or through ICTs might not involve an imbalance of power or repetition, which are the core criteria for the traditional face-to-face victimization definition [7]. Furthermore, the behaviors used to harm someone are not always equivalent online and offline, such as hacking someone’s Facebook account or happy slapping (i.e., taking a video or picture of someone randomly slapping someone else and then uploading it online) [8].

It is important to study cyber victimization because victims report psychological and behavioral consequences [9,10,11,12]. Much of the research on cyber victimization has focused on its associations with psychological consequences, including depression, anxiety, and loneliness [13,14,15]. Furthermore, victims often have school functioning difficulties and problems with academic performance, suggesting that their online experiences interfere with their offline behaviors [14,16,17]. The findings of previous research underscore the importance of examining the psychological consequences of cyber victimization. In addition, these studies also highlight the importance of determining variables that might influence the psychological consequences associated with cyber victimization, particularly variables that might worsen these relationships.

Few studies have documented the cyber victimization experiences of minority adolescents. In one study, Wang et al. [18] found that black students had the highest rates of cyberbullying (10.9%), followed by Latinx students (9.6%), other race/ethnic backgrounds (7.3%), and white students (6.7%). Similarly, Kessel Schneider and colleagues [19] found that non-white students reported greater cyber victimization (8.4%) when compared to white students (5.7%). Various researchers [2,10,20,21,22] have further examined different patterns of cyber victimization experiences among ethnic minority adolescents. Other than studies focusing on general patterns of cyber victimization experiences, little attention has been given to potential differences in psychological consequences resulting from such experiences among adolescents of different ethnicities. In one study on this topic, Edwards et al. [23] examined depression symptoms, suicidal ideation, and suicidal attempts following cyber victimization among black, Latinx, Asian, and white adolescents. Their findings revealed that Latinx adolescents were somewhat more vulnerable to depressive symptoms and suicidal ideation, while Asian and white adolescents were less vulnerable. Although they acknowledged that such differences were small, researchers might want to consider including ethnicity when examining the psychological consequences of cyber victimization.

### 1.2. School-Belongingness

School-belongingness is defined as students’ beliefs and feeling that they are personally accepted, respected, included, and supported by others in the school social environment [24]. Consequently, school-belongingness goes beyond students’ enrollment at school and encompasses the extent to which they have established social bonds with their teachers and peers and perceive the school as protective [25]. Racial and ethnic differences have been found in adolescents’ sense of school-belongingness, with findings revealing that marginalized minority adolescents, such as African Americans, American Indians, and Latinx adolescents, report lower school belongingness when compared to Asian American and European American adolescents [3].

Research concludes that lower school-belongingness relates to higher cyber victimization [26,27,28]. Negative psychological functioning (e.g., depression, anxiety, loneliness) is also correlated positively with low school-belongingness [29,30,31,32]. However, it is unclear whether school-belongingness moderates the associations between cyber victimization and psychological consequences. Available research has revealed that school-belongingness moderated the relationship between peer attachment and school misconduct [33], as well as moderated the association between academic stress and depression [34]. Based on the available research findings on the moderation of school-belongingness, it might be reasonable to expect that this variable might also moderate the relationship between cyber victimization and depression, anxiety, and loneliness. Incidences of cyber victimization carry over into the school environment as such behaviors usually involve peers at the same school [35]. Given the propensity for cyber victimization to involve peers from the same school, it is likely that such experiences influence school-belongingness and subsequent psychological consequences. Such a proposal might be likely because school-belongingness involves feelings of social support and bonding with individuals in the school environment. Therefore, school-belongingness might exacerbate or mitigate the negative consequences of cyber victimization.

### 1.3. The Present Study

The purpose of the present study was to investigate the potential moderating effect of ethnicity in the associations among cyber victimization, school-belongingness, and negative psychological consequences (i.e., depression, anxiety, loneliness). This involved examining three-way interactions among ethnicity (Latinxs and white), cyber victimization, and school-belongingness. Another aim of the study was to examine these associations over one year, by assessing the variables in 7th grade (Time 1) and then assessing depression, anxiety, and loneliness in 8th grade (Time 2). The following hypotheses were proposed to address the study’s aim:

Hypothesis 1: The association between cyber victimization and Time 2 depression, anxiety, and loneliness will be more positive for Latinx adolescents than white adolescents when they have lower levels of school-belongingness.

Hypothesis 2: The association between cyber victimization and Time 2 depression, anxiety, and loneliness will be less positive for Latinx adolescents than white adolescents when they have higher levels of school-belongingness.

## 2. Methods

### 2.1. Participants

Participants were 416 adolescents (46% female; *M* age = 13.89, *SD* = .41) from one Midwestern middle school in the United States, with an almost equal mix of Latinx (43%; *n* = 202; 53% girls) and white (44%; *n* = 214; 49% girls) adolescents. Adolescents were in the 8th grade at the end of the study. Other ethnicities were not included in the study due to low frequency rates (7% for Asian; 6% for Black/African American). The school is located in a predominantly middle-class neighborhood. Approximately 33% of students from the school received a free or reduced-cost lunch. Family composition consisted of two-parent households (61%), single-parent households (30%), or living with a legal guardian (9%). The median income of the families was $51,376.

### 2.2. Procedures

Institutional approval was obtained for this study, with protocol number MW080210PSY. Five schools were picked at random from a list of over 153 middle schools located in the Midwestern United States. Recruitment emails were sent to the school principals. Of the five principals emailed, three indicated that they had pre-existing engagements, one never responded, and the other agreed to meet with the principal investigator of the study. After the meeting, the school principal agreed to allow students to participate. A meeting was then conducted among the principal investigator, school principal, and 7th and 8th grade teachers. The purpose of the meetings was to describe the purpose of the study, time commitment of the study, and what adolescents would be expected to do if they were to participate in the study. Announcements were made to 7th grade classes to describe the study and how adolescents could participate. Parental permission slips were sent home to adolescents’ parents. The permission slip was available in English and Spanish and described the study and what adolescents would be asked if their child was to participate. A sheet asking for family income and family structure was included with the permission slip. There were 499 parental permission slips distributed, and of these, 423 were returned to the school with permission, 10 were returned without permission, and the rest were never returned. During the 7th grade (Time 1), adolescents completed measures on cyber victimization, face-to-face victimization, depression, anxiety, loneliness, and school-belongingness. The total number of adolescents who participated at Time 1 was 422, as one adolescent was absent on the day of data collection.

One year later, during the 8th grade (Time 2), a reminder letter was sent home with the 422 adolescents from Time 1. The purpose of the letter was to remind parents/guardians about the study their child participated in one year earlier. If parents/guardians did not want their child to participate in the study again, they were asked to write their child’s name on the sheet and return it to their child’s school. No reminder letters were returned. Of the 422 participants from Time 1, five participants had moved away and one was unavailable during data collection. At Time 2, adolescents completed measures on depression, anxiety, and loneliness measures.

### 2.3. Measures

#### 2.3.1. Cyber Victimization

To assess adolescents experience of victimization online or through text messages, they answered nine items on a scale of 1 (*not at all*) to 5 (*all of the time*) [35]. Adolescents were asked to indicate how often they experienced these behaviors within the current school year. Sample items included: Someone gossiped about me online or through text messages, someone sent me a nasty message online or through text messages, and someone insulted me online or through text messages. This measure was administered at Time 1 only. The nine items were combined to form a final score of cyber victimization. Cronbach’s alpha was 0.91.

#### 2.3.2. Face-to-Face Victimization

Adolescents rated 12 items on a scale of 1 (*not at all*) to 5 (*all of the time*) regarding how often they experienced face-to-face bullying victimization within the current school year [36]. Sample items included: Someone gossiped about me, someone spread a rumor about me, and someone hit, kicked, or punched me. This measure was administered at Time 1 only; all 12 items were combined to form a final score of face-to-face victimization, with a Cronbach’s alpha of 0.93.

#### 2.3.3. Depression

The Center for Epidemiological Studies Depression Scale was used to measure adolescents’ depressive symptoms [37]. Adolescents rated 20 items on a scale of 0 (*rarely or none of the time*) to 3 (*most or all of the time*). Sample items included: I was bothered by things that usually don’t bother me and I did not feel like eating, my appetite was poor. Items were combined to form final scores of depression at Time 1 and Time 2. Cronbach’s alphas were 0.92 at Time 1 and 0.91 at Time 2.

#### 2.3.4. Anxiety

The Multidimensional Anxiety Scale for Children was used to assess adolescents’ anxiety symptoms [38]. The 39 items were rated on a scale of 0 (never true about me) to 3 (often true about me. A sample item included: I get scared when my parents go away. The items were combined to form final scores of anxiety at Time 1 and Time 2. Cronbach’s alphas were 0.91 at Time 1 and Time 2.

#### 2.3.5. Loneliness

Adolescents completed the Revised UCLA Loneliness Scale to assess their symptoms of loneliness [39]. They rated 20 items on a scale of 1 (never) to 4 (*often*), with a sample item of “I feel isolated from others”. The items were combined to form final scores at Time 1 and 2, with Cronbach’s alphas of 0.92 at Time 1 and 0.93 at Time 2.

#### 2.3.6. School-Belongingness

Adolescents answered 18 items regarding their feelings about school in general, what they think about classes, teachers, and other students, and their overall sense of belongingness in the school environment [24]. Items were answered on a scale of 1 (*not at all true*) to 5 (*completely true*). Items were combined to form a final score of school belongingness. Sample items included: The teachers here respect me and other students here like me the way I am. This measure was administered at Time 1 only. Cronbach’s alpha was 0.91.

### 2.4. Analytic Plan

Multiple hierarchical regression analyses were conducted with Time 2 depression, loneliness, and anxiety as the dependent variables. Block 1 included ethnicity, gender, and Time 1 face-to-face victimization, as well as Time 1 depression, loneliness, and anxiety. Block 2 included Time 1 cyber victimization. Block 3 included Time 1 school-belongingness. Block 4 included two-way interactions between Time 1 cyber victimization and Time 1 school-belongingness, Time 1 cyber victimization and ethnicity, and Time 1 school-belongingness and ethnicity. Block 5 included a three-way interaction among Time 1 cyber victimization, Time 1 school-belongingness, and ethnicity. Time 1 depression, loneliness, and anxiety were included in the model to control for these psychological consequences. The variance inflation factor was not greater than 1.10 for any of the variables, indicating that multicollinearity was not an issue. Significant interactions were probed using the Interaction program [40]. This program provides statistics regarding the simple slopes at the +1 standard deviation (*SD*), −1 *SD*, and mean, as well as the significance of these slopes and a graphical representation of the interaction.

## 3. Results

Correlations were examined among the variables in the study (see Table 1). The findings revealed that cyber victimization was related positively to face-to-face victimization, as well as Time 1 and Time 2 depression, loneliness, and anxiety. Cyber victimization was related negatively to school-belongingness. School belongingness was related negatively to Time 1 and Time 2 depression, loneliness, and anxiety. Time 1 and Time 2 depression, loneliness, and anxiety were associated positively with each other, except for Time 2 depression and Time 1 anxiety, and Time 1 loneliness and Time 2 anxiety.

Block 5 was the highest significant block for Time 2 depression and anxiety, while Block 4 was the highest significant block for Time 2 loneliness (see Table 2). Cyber victimization was related positively to Time 2 depression, anxiety, and loneliness, while school-belongingness was associated negatively to these variables. Face-to-face victimization was associated positively with Time 2 depression, anxiety, and loneliness. Time 2 depression was related positively to Time 1 depression, with similar patterns among Time 1 and Time 2 anxiety, and Time 1 and Time 2 loneliness.

**Figure 1:**
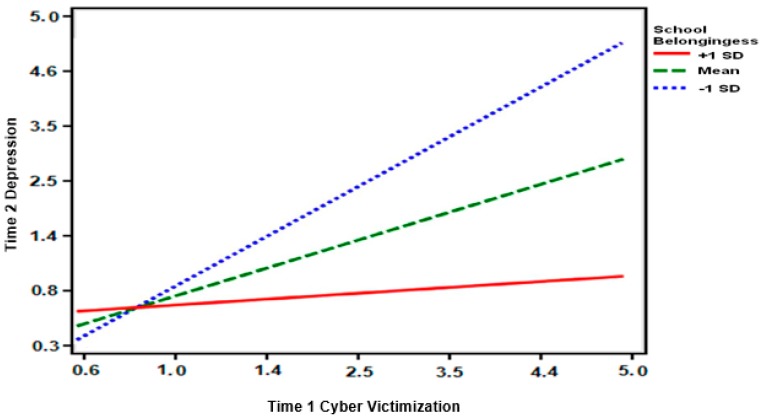
Graphical representation of the interaction between cyber victimization and school-belongingness for depression among Latinx adolescents.

**Figure 2:**
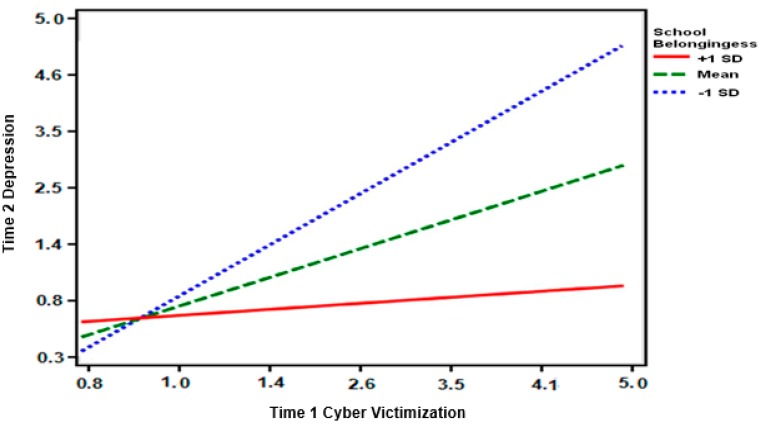
Graphical representation of the interaction between cyber victimization and school-belongingness for anxiety among Latinx adolescents.

For the model with Time 2 loneliness, the two-way interaction between cyber victimization and school-belongingness was significant (see Figure 1). Low levels of school-belonging increased the association between cyber victimization and Time 2 loneliness. For the models with Time 2 depression and anxiety, the three-way interactions among cyber victimization, school-belongingness, and ethnicity were significant (see Figure 2). To probe the interaction further, the interactions between cyber victimization and school-belongingness were examined separately for Latinx and white adolescents. The interactions were not significant for white adolescents. For Latinx adolescents, the interactions were significant for both Time 2 depression (see Figure 1) and anxiety (see Figure 2). Time 2 depression and cyber victimization were more strongly associated at lower levels of school-belongingness for Latinx adolescents. Similar patterns were found for Time 2 anxiety, providing partial support for Hypothesis 1 because the interaction for Time 2 loneliness was not found to differ for Latinx and white adolescents. Hypothesis 2 was not supported as none of the simple slopes were significant for high levels of school-belongingness.

## 4. Discussion

The purpose of this one-year longitudinal study was to investigate the moderating effect of adolescents’ ethnicity on the relationships among cyber victimization, school-belongingness, and psychological consequences (i.e., depression, loneliness, anxiety). The findings from this study aid our understanding of the impact of ethnicity on the cyber victimization experiences among Latinx and white adolescents, as well as contribute to the growing body of research on how the school context, particularly adolescents’ feelings of school-belongingness, influences adolescents’ psychological consequences following their experience of cyber victimization. This research also expands the present research on the cyber victimization experiences of ethnic minority adolescents, as much of the literature focuses on prevalence rates of this experience. Expanding the previous research on cyber victimization and ethnic minorities [18,19,20,21,22], the findings of this present study revealed that the association between cyber victimization and depression and anxiety were more positive for Latinx adolescents with low levels of school-belongingness.

Copious attention has been given to the psychological consequences associated with cyber victimization, including depression, anxiety, and loneliness [13,14,15]. Although some attention has been given to the cyber victimization experiences of Latinx adolescents, particularly the frequency rates of this experience, there is still a gap in the literature. Few studies involve longitudinal designs or consider how school-related experiences might influence the negative consequences associated with cyber victimization. We hypothesized that low levels of Time 1 school-belongingness would increase the positive association between Time 1 cyber victimization and Time 2 loneliness, and that these associations would be different for Latinx adolescents versus white adolescents (Hypothesis 1). Our findings only partially supported this hypothesis as we did not find support that such associations were different for Latinx and white adolescents, although the two-way interaction between cyber victimization and school-belongingness was significant for all adolescents. A potential explanation for these findings might be that adolescents with low school-belongingness might be at risk for loneliness regardless of their ethnicity. School-belongingness involves “relatedness” and the secure and satisfying connections with others [41,42]. It implies that the individual feels supported and connected in the school context. Loneliness results from being dissatisfied with relationships with others [43]. Therefore, adolescents, regardless of ethnicity, might feel lonely and experiencing lower levels of school-belongingness and cyber victimization, both of which relate to loneliness, might further increase their vulnerability to loneliness.

We did find some support for Hypothesis 1 when considering Time 2 depression and anxiety. Although the two-way relationship between cyber victimization and low school-belongingness was supported for all adolescents, this association was found to be especially strong for Latinx adolescents. Available research suggests that ethnic minority adolescents, including Latinx adolescents, experience higher cyber victimization than white adolescents [18]. Given Latinx adolescents greater experience with cyber victimization, they might be at particular risk for depression and anxiety resulting from this experience. Furthermore, Latinx adolescents report lower levels of school-belongingness more generally, which might further reduce their connection with their peers and contribute to their experience of depression and anxiety [3]. Low school-belongingness indicates that these adolescents feel as if they are not supported by their teachers and peers, potentially reducing their ability to effectively cope with cyber victimization and increasing their vulnerability to depression and anxiety.

We expected that high levels of school-belongingness would moderate the associations between cyber victimization and Time 2 depression, anxiety, and loneliness for Latinx adolescents (Hypothesis 2). The findings were not found for either Latinx or white adolescents. A lack of significant results regarding the potential protective function of high school-belongingness is puzzling, given the existing literature on this topic. Musthafa et al. [34] and Liu and Li [35] found support for the buffering effect of high school-belongingness for reducing school behavioral misconduct and academic stress. These researchers focused on school-related outcomes, while our focus is more generally on depression, anxiety, and loneliness, which might explain differences in the findings of the present student with the literature. Furthermore, school-belongingness is unrelated to friendship quality or increases in social networks of friends [44]. It might be likely that friends are more effective as a source of social support used for coping with cyber victimization and reducing the negative consequences of cyber victimization than making students feel generally connected to the school environment. We are not suggesting that high school-belongingness does not imply social support for various other stressors in adolescents’ lives, but that it might be less effective than using friends as a source of social support for coping with cyber victimization. More research is needed to understand this complex relationship.

### 4.1. Limitations and Future Directions

The longitudinal nature of this study is an important contribution to the literature but there are a few limitations and future research directions that need to be discussed. First, the study utilized self-reports of cyber victimization, school-belongingness, depression, anxiety, and loneliness. Research incorporating peer-nominations or peer-ratings, as well as teacher-reports of cyber victimization, depression, anxiety, and loneliness might be a fruitful direction for follow-up research. We assessed cyber victimization and school-belongingness at one time point in the 7th grade. Such a methodology limits our understanding of the temporal ordering of the variables examined in this study. Future research should focus on multiple assessments with long-term designs to better understand the associations of the variables. Although the ethnic breakdown of adolescents from the school allowed us to compare Latinx and white adolescents, we were not able to investigate other ethnicities, due to low rates. In addition, Latinx adolescents do not represent a homogenous group of individuals. Instead, these adolescents’ families might originate from different Spanish-speaking countries in which cultural practices might vary. Therefore, future research might benefit from examining Latinx adolescents from various countries. A final limitation is that the study involved adolescents from one school. Therefore, generalizability of the findings might not apply to other samples.

## 5. Conclusions

The present study is one of the few longitudinal studies focused on investigating the moderating effect of ethnicity in the associations among cyber victimization, school-belongingness, and psychological consequences, including depression, loneliness, and anxiety. It is among the few studies to focus on comparing the cyber victimization experiences between Latinx and white adolescents; such a focus adds to a body of literature that has mostly focused on the prevalence rates of cyber victimization for ethnic minority adolescents. Adolescents’ low school-belongingness worsens the negative consequences associated with cyber victimization, particularly for Latinx adolescents; this finding has important implications for understanding the interaction between an out-of-school experience (i.e., cyber victimization) and the school environment. Our findings further suggest the importance of a multi-pronged solution focused on raising awareness of cyber victimization not only in adolescents’ homes or through their mobile devices but also in their schools. This study has the potential to inform school personnel about the implications of low school-belongingness among Latinx and white adolescents, and to indicate the need to promote a sense of a caring and relatable community within the school environment.

## Figures and Tables

**Table 1:** Correlation among the study’s variables.

Variable	1	2	3	4	5	6	7	8	9
1. Face-to-face Victimization	---								
2. Cyber Victimization	0.43 ***	---							
3. School-Belongingness	−0.26 **	−0.27 **	---						
4. Time 1 Depression	0.30 ***	0.31 ***	−0.20 *	---					
5. Time 2 Depression	0.29 ***	0.29 ***	−0.22 *	0.41 ***	---				
6. Time 1 Loneliness	0.26 **	0.25 **	−0.15	0.25 **	0.26 **	---			
7. Time 2 Loneliness	0.23 *	0.22 *	−0.19 *	0.22 *	0.22 *	0.38 ***	---		
8. Time 1 Anxiety	0.26 **	0.27 **	−0.16	0.23 *	0.15	0.20 *	0.19 *	---	
9. Time 2 Anxiety	0.25 **	0.26 **	−0.20 *	0.20 *	0.20 *	0.16	0.18 *	0.39 ***	---

* *p* < 0.05. ** *p* < 0.01. *** *p* < 0.001.

**Table 2:** Predicting depression, loneliness, and anxiety from gender, ethnicity, face-to-face victimization, and school-belongingness.

Variable	Time 2 Depression			Time 2 Loneliness			Time 2 Anxiety		
	β	R^2^	Δ R^2^	β	R^2^	Δ R^2^	β	R^2^	Δ R^2^
Block 1		.15	.15 ***		.12	.12 ***		.10	.10 ***
Gender	0.08			0.11			0.09		
Ethnicity	0.05			0.06			0.08		
Face-to-face Victimization	0.18 *			0.16			0.16		
Adjustment	0.31 ***			0.29 ***			0.27 **		
Block 2		.25	.10 ***		.20	.08 ***		.18	.08 ***
Gender	0.07			0.11			0.08		
Ethnicity	0.04			0.06			0.06		
Face-to-face Victimization	0.16			0.14			0.12		
Adjustment	0.30 ***			0.27 **			0.25 **		
Cyber Victimization (CV)	0.27 **			0.25 **			0.21 **		
Block 3		.31	.06 ***		.25	.05 ***		.24	.06 ***
Gender	0.07			0.10			0.07		
Ethnicity	0.04			0.06			0.05		
Face-to-face Victimization	0.15			0.13			0.12		
Adjustment	0.28 **			0.26 **			0.22 **		
CV	0.25 **			0.23 **			0.20 **		
School Belongingness (SB)	−0.27**			−0.26 **			−0.25 **		
Block 4		.37	.06 ***		.31	.06 ***		.30	.06 ***
Gender	0.06			0.09			0.08		
Ethnicity	0.03			0.06			0.03		
Face-to-face Victimization	0.13			0.10			0.12		
Adjustment	0.26 **			0.23 **			0.20 *		
CV	0.24 **			0.22 **			0.18 *		
SB	−0.26 **			−0.26 **			−0.25 **		
CV × SB	0.17 *			0.15 *			0.14 *		
CV × Ethnicity	0.06			0.02			0.01		
SB × Ethnicity	0.02			0.03			0.02		
Block 5		.42	.05 ***		.31	.01		.35	.05 ***
Gender	0.11			0.10			0.11		
Ethnicity	0.03			0.05			0.04		
Face-to-face Victimization	0.11			0.06			0.10		
Adjustment	0.23 **			0.21			0.16 *		
CV	0.21 **			0.20			0.18 *		
SB	−0.23 **			−0.24			−0.21 **		
CV × SB	0.16 *			0.15			0.13 *		
CV × Ethnicity	0.05			0.04			0.01		
SB × Ethnicity	0.03			0.04			0.03		
CV × SB x Ethnicity	0.29 ***			0.07			0.27 ***		

* *p* < 0.05. ** *p* < 0.01. *** *p* < 0.001.
